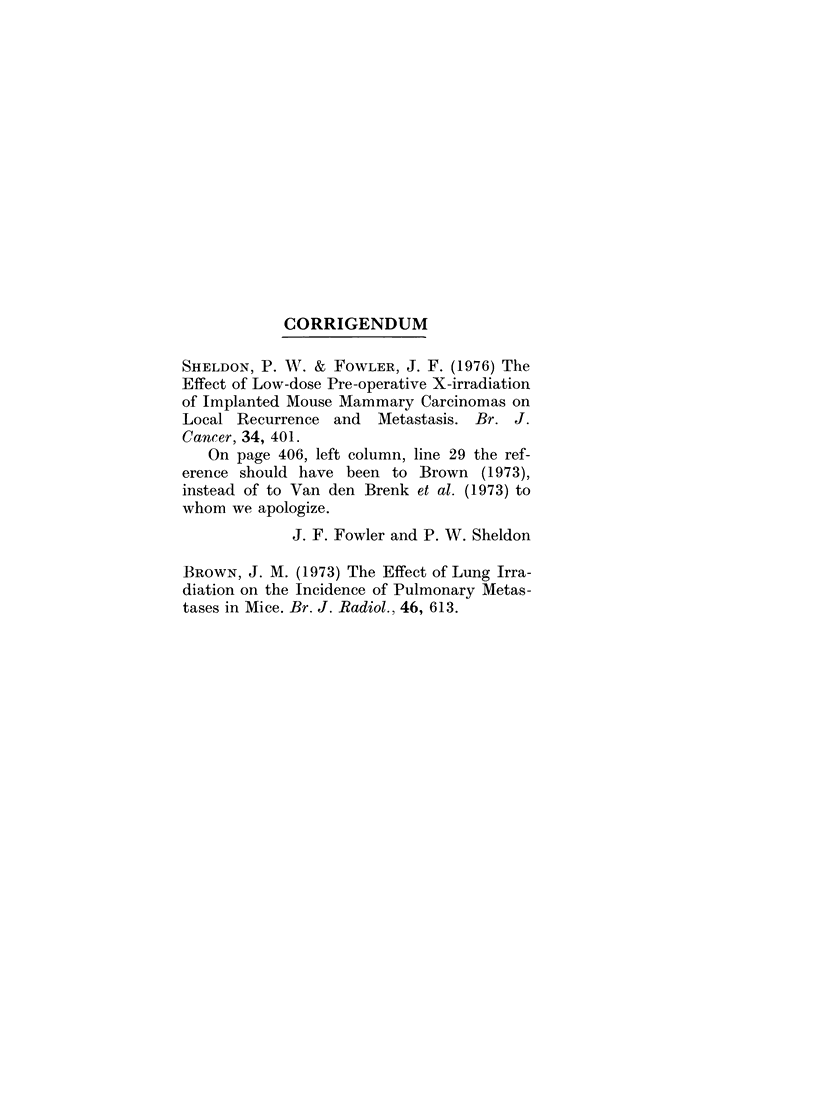# Corrigendum

**Published:** 1976-10

**Authors:** 


Brown, J. M. (1973) The Effect of Lung Irradiation on the Incidence of Pulmonary Metastases in Mice. *Br. J. Radiol.*, **46,** 613.